# Geographical Variation in the Use of Oral Anticoagulation and Clinical Outcomes among Patients with Atrial Fibrillation in Denmark, Sweden, and Finland

**DOI:** 10.1055/a-2080-6171

**Published:** 2023-06-06

**Authors:** Lars Frost, Olli Halminen, Mika Lehto, K E. Juhani Airaksinen, Tomas Andersson, Per Wändell, Martin Holzmann, Pia Cordsen, Nicklas Vinter, Søren Paaske Johnsen

**Affiliations:** 1 Diagnostic Centre, Silkeborg Regional Hospital, Silkeborg, Denmark; 2 Department of Clinical Medicine, Aarhus University, Aarhus, Denmark; 3 Department of Industrial Engineering and Management, Aalto University, Espoo, Finland; 4 Department of Internal Medicine, Hospital District of Helsinki and Uusimaa, Lohja Hospital, Lohja, Finland; 5 Heart and Lung Center, Helsinki University Hospital and University of Helsinki, Helsinki, Finland; 6 Turku University Hospital, University of Turku, Turku, Finland; 7 Institute of Environmental Medicine, Karolinska Institutet, Stockholm, Sweden; 8 Division of Family Medicine and Primary Care, Department of Neurobiology, Care Sciences and Society, Karolinska Institutet, Huddinge, Sweden; 9 Functional Area of Emergency Medicine, Karolinska University Hospital, Stockholm, Sweden; 10 Department of Internal Medicine Solna, Karolinska Institutet, Stockholm, Sweden; 11 Danish Center for Clinical Health Services Research, Aalborg University, Aalborg, Denmark

**Keywords:** atrial fibrillation, anticoagulation, bleed, geographical variation, stroke

## Abstract

**Background** Geographical mapping of variations in the treatment and outcomes of a disease is a valuable tool for identifying inequity. We examined international and intranational variations in initiating oral anticoagulation (OAC) therapy and clinical outcomes among patients with atrial fibrillation (AF) in Nordic countries. We also tracked real-world trends in initiating OAC and the clinical outcomes.

**Methods** We conducted a registry-based multinational cohort study of OAC-naive patients with an incident hospital diagnosis of AF in Denmark ( *N* = 61,345), Sweden ( *N* = 124,120), and Finland ( *N* = 59,855) and a CHA _2_ DS _2_ -VASc score of ≥1 in men and ≥2 in women between 2012 and 2017. Initiation of OAC therapy was defined as dispensing at least one prescription between 90 days before and 90 days after the AF diagnosis. Clinical outcomes included ischemic stroke, intracerebral hemorrhage, intracranial bleeding, other major bleeding, and all-cause mortality.

**Results** The proportion of patients initiating OAC therapy ranged from 67.7% (95% CI: 67.5–68.0) in Sweden to 69.6% (95% CI: 69.2–70.0) in Finland, with intranational variation. The 1-year risk of stroke varied from 1.9% (95% CI: 1.8–2.0) in Sweden and Finland to 2.3% (95% CI: 2.2–2.4) in Denmark, with intranational variation. The initiation of OAC therapy increased with a preference for direct oral anticoagulants over warfarin. The risk of ischemic stroke decreased with no increase in intracranial and intracerebral bleeding.

**Conclusion** We documented inter- and intranational variation in initiating OAC therapy and clinical outcomes across Nordic countries. Adherence to structured care of patients with AF could reduce future variation.

## Introduction

The lifetime risk of atrial fibrillation (AF) is one in three, 1 and AF is a well-recognized risk factor for heart failure, stroke, peripheral arterial thromboembolism, dementia, and death. Oral anticoagulation (OAC) has been considered the state of the art for the prevention of stroke and peripheral thromboembolism in patients with AF for more than three decades.

In 2012, in Europe, direct oral anticoagulants (DOACs) started to be marketed. This new drug class complemented warfarin as an OAC treatment to prevent stroke and systemic embolism in patients with AF. A systematic meta-analysis of DOACs versus warfarin documented the superiority of DOACs over warfarin, with a 19% (relative risk: 0.81, 95% confidence interval [CI] 0.73–0.91; *p* < 0.0001) reduced risk of stroke or systemic embolic events, mainly driven by a reduction in hemorrhagic stroke (0.49, 0.38–0.64; *p* < 0.0001). DOACs also reduced all-cause mortality (0.90, 0.85–0.95; *p* = 0.0003) and intracranial hemorrhage (0.48, 0.39–0.59; *p* < 0.0001. 2

A systematic review and meta-analysis of worldwide trends of AOC use among patients with AF from 2010 to 2018 documented that the proportion of OAC users worldwide almost doubled following the introduction of DOACs, although one out of four patients eligible for OAC was not anticoagulated. 3 Previous studies have also documented an increasing uptake of OACs in the pre-DOAC era. 4 5 The increased uptake of OACs prompted by the introduction of DOACs has been associated with a decrease in the risk of stroke among patients with AF. 4 5 6 7 8 9 10 11 12 13 This evolution of increasing usage of OACs and decreasing risk of stroke has been reported in several countries, including the United States, 4 Denmark, 5 England, 10 Sweden, 6 Germany, 7 Korea, 8 Italy, 9 and Japan. 11

Denmark, Sweden, and Finland have national administrative registries holding information about hospitalizations, medical treatment, and mortality, which provide unique opportunities for examining international and intranational variations in the initiation of OAC in the entire population of patients with a hospital diagnosis of AF. In this study on patients with incident AF, we report on international and intranational variations in the use and preferences of OACs and clinical outcomes: ischemic stroke, intracerebral hemorrhage, intracranial bleeding, other major bleeding, and all-cause mortality. We also report real-world trends in the initiation of OACs and clinical outcomes.

## Methods

### Setting

The populations of Denmark (5.8 million), Sweden (10.0 million), and Finland (5.5 million) sum up to 21.3 million inhabitants. The health care systems in these countries have many similarities, including tax-paid health care, with only a very small amount of user payments per visit for medical care in Sweden and Finland (∼ 25€ per visit and a maximum of 130€ per year in Sweden, and 40€ per visit and a maximum of 690€ per year in Finland) and none in Denmark. In these countries, there is a partial reimbursement of consumer costs of medical treatment. All three countries have complete and updated information on citizens' vital and emigration status, and all maintain registries with information on hospitalizations and pharmacy-dispensed medication. A unique civil registration number allocated to each individual enables linkage among the nationwide registries within each country.

### Data Sources

Nationwide patient registries were used and cross-linked with nationwide prescribed drug registries and civil registration systems. National patient registries were established in 1977 in Denmark, 1987 in Sweden, and 1967 in Finland, and they contain prospectively registered data on hospital inpatients and outpatients. The data include individual-level information on dates of admission and discharge, surgical procedures performed, and one primary and multiple secondary diagnoses per discharge. The coding of diagnoses followed the International Classification of Diseases, 10th edition (ICD-10). The nationwide prescription registries were established in 1995 in Denmark, 2005 in Sweden, and 1994 in Finland, and they contain individual-level data on all dispensed prescriptions. The coding follows the Anatomical Therapeutic Chemical (ATC) Classification System. The civil registration systems contain individual-level information on sex, date of birth, migration status and dates, and vital status and date of death.

### Design and Study Populations

We conducted country-specific nationwide cohort studies. The baseline was on the day of diagnosis of AF, and the follow-up period was up to and including 2018 in Denmark and Finland and up to and including 2017 in Sweden.

We included all patients aged ≥40 and <90 years with a first-time diagnosis of AF reported from a hospital ward or a hospital outpatient clinic to the respective national patient registries. The inclusion period was between January 1, 2012, and December 31, 2017, in Denmark and Finland and January 1, 2012, and December 31, 2016, in Sweden. A diagnosis of AF (primary or secondary diagnosis, including in-hospital outpatient clinics) was identified using the ICD-10 code I48. The date of diagnosis was on the discharge day for inpatients or the day of first contact for outpatients.

The exclusion criteria were:

- Patients whose first-time diagnosis of AF was valvular AF (AF with mitral stenosis or mechanical prosthetic heart valves; Supplementary Table S1, available online only ). 14
- Patients with less than 5 years of a complete look-back history in the national registries.
- Male patients with a modified CHA _2_ DS _2_ -VASc score of 0 and female patients with a modified CHA _2_ DS _2_ -VASc score of 1 (definition given in Supplementary Table S2, available online only ).
- Patients with civil registration status classified as inactive or disappeared in all or part of the study period.
- Patients redeeming a prescription for an OAC on day -91 or prior to the day of the AF diagnosis ( Supplementary Fig. S1, available online only ).
- Patients with a history of ischemic stroke, defined as a diagnosis on or before the baseline date (see also Supplementary Table S3, available online only ).

### Ascertainment of Regions

Information on residential regions/counties was identified in the national civil registration systems. Denmark has 5 regions, Sweden has 21 counties, and Finland has 5 university hospital regions.

### OAC Initiation

We defined initiation of OAC therapy as filling at least one prescription for OAC therapy between 90 days before and 90 days after baseline. In a sensitivity analysis, we increased this time window for initiating OAC therapy to 180 days before and 90 days after baseline. In another sensitivity analysis, we restricted the analysis to patients with a primary diagnosis of AF and used the time window for initiating OAC therapy from 90 days before to 90 days after the AF diagnosis. The national prescription registries were used to identify the OAC therapy using Anatomical Therapeutic Chemical classification (ATC) codes ( Supplementary Table S4, available online only ).

### Clinical Outcomes and Mortality

We computed the risk of ischemic stroke, intracerebral hemorrhage, intracranial bleeding, other major bleeding, and all-cause mortality through 1 year after baseline. The diagnoses were identified as inpatient and outpatient contacts from the national patient registries using ICD-10 ( Supplementary Table S3, available online only ). We considered both primary and secondary diagnoses.

### Covariates

Covariate information was retrieved at baseline and included the following comorbidities: abnormal liver function, abnormal renal function, alcohol-related disease, bleeding, congestive heart failure, diabetes, hypertension, thromboembolism, stroke, and vascular disease. The national patient registries provided information on diagnoses using ICD-10. ICD-10 was implemented in Denmark in 1994, in Sweden in 1997, and in Finland in 1996. For the history of AF and comorbidities, we used a maximal look-back period in ICD-10. For some conditions, we also used information on medication to define conditions using the prescription registries ( Supplementary Table S2, available online only ). We only considered medication redeemed in a window of 1 year before baseline.

We computed a modified CHA _2_ DS _2_ -VASc score and a modified HAS-BLED score for each patient. Because information on the international normalized ratio (INR) was unavailable, the HAS-BLED was “modified.” Supplementary Table S2 (available online only) shows the definition of the modified CHA _2_ DS _2_ -VASc score, and Supplementary Table S5 (available online only) shows the definition of the modified HAS-BLED score.

Concomitant medication at baseline encompassed antiplatelet drugs, NSAIDs (nonsteroidal anti-inflammatory drugs), and statins. Medication was identified from the national prescription registries using ATC codes ( Supplementary Table S6, available online only ).

### Statistical Analyses

The data were distributed so that each nation kept their own dataset. We applied a common distributed data model and detailed analysis plan to ensure that all variables were defined in the same way across the countries and shared a syntax for the analyses.

We estimated the period prevalence of OAC initiators as the number of initiators divided by the number of patients with AF. To estimate a relative measure of initiation by region, we used Poisson regression. Adjustments followed Model 1 and Model 2 as specified in Supplementary Table S7 (available online only) . We used the Aalen-Johansen estimator with death as a competing event to compute the 1-year cumulative incidence (95% CI) of the clinical outcomes for the entire nation and by region. We computed the pseudo-observations for each outcome in generalized linear models to estimate the relative risks and compare the risk for each of the clinical outcomes across regions. 15 16 17 Adjustments followed Model 1 and Model 2 as specified in Supplementary Table S7 (available online only) . We used the Kaplan‒Meier estimator to compute the cumulative mortality (95% CI) 1 year after baseline for the entire nation and by region. We computed the pseudo-observations for mortality in generalized linear models to estimate the relative risk and compare the risk across regions. Nation-specific estimates of initiation, clinical outcomes, and mortality were compared across countries based on 95% CIs. No formal statistical test was applied. We examined the temporal trends in the outcomes by computing the cause-specific hazard ratios and associated Wald tests for the calendar year as a continuous variable using Cox regression models. In the analyses of the outcomes after AF, the follow-up ended at the date of the specified outcome, emigration, death, or the end of follow-up, whichever came first.

For all statistical analyses, we used Stata version 16.1, StataCorp LLC in Denmark; R version 4.1.1 in Sweden; and IBM SPSS Statistics version 27 for descriptive statistics and Stata version 17, StataCorp LLC, for statistical analyses in Finland.

### Ethics

In Denmark, registry-based studies do not require ethics approval. In Sweden, the study was approved by the Regional Ethical Review Board in Stockholm (dnr: 3510/2019). Only aggregated anonymous data were exported. In Finland, no patient consent is needed according to Finnish legislation. This study was approved by the Ethics Committee of the Medical Faculty of Helsinki University, Helsinki, Finland, and research permission was granted by the Helsinki University Hospital.

## Results

### Study Population Characteristics

Our study cohorts included 61,345 patients with incident AF in Denmark, 124,120 in Sweden, and 59,855 in Finland with a modified CHA _2_ DS _2_ -VASc score ≥1 in men and ≥2 in women and no history of stroke over the study inclusion period from 2012 to 2017. A flowchart of the study cohorts is shown in Supplementary Table S8 .

The national study cohorts are characterized in Table 1 . The median age was 73 to 75 years. The proportion of men was 55% in Denmark and Sweden and 50% in Finland. We noted variations among the countries in the proportion of comorbidities and concomitant medication, notably a higher prevalence of a history of myocardial infarction in Sweden, a higher prevalence of hypertension in Denmark, and a higher use of antiplatelet medication in Denmark and Sweden before AF was diagnosed ( Table 1 ).

**Table 1:** Baseline characteristics of patients with atrial fibrillation in Denmark, Sweden, and Finland

Characteristics	Denmark *n* = 61,345	Sweden *n* = 124,120	Finland *n* = 59,855
Men, % ( *n* )	55.4 (33,989)	55.3 (68,627)	49.5 (29,599)
Age, median (IQR), year	73 (66–80)	75 (69–82)	74 (67–81)
Baseline year, % ( *n* )			
2012	16.8 (10,293)	16.9 (20,926)	16.7 (10,009)
2013	17.2 (10,565)	17.0 (21,149)	17.2 (10,299)
2014	16.6 (10,211)	16.6 (20,607)	17.0 (10,147)
2015	16.8 (10,316)	16.6 (20,544)	15.9 (9,500)
2016	16.5 (10,111)	16.6 (20,545)	16.9 (10,133)
2017	16.1 (9,849)	16.4 (20,349)	16.3 (9,767)
History of comorbidity			
Abnormal liver function	2.0 (1,198)	2.0 (2,466)	1.3 (791)
Abnormal renal function	6.0 (3,653)	6.0 (7,440)	2.4 (1,434)
Alcohol-related disease	4.0 (2,444)	6.3 (7,875)	3.1 (1,834)
Bleeding	12.3 (7,531)	13.6 (16,837)	7.9 (4,744)
Congestive heart failure	13.7 (8,401)	13.7 (17,046)	5.4 (3,217)
Diabetes mellitus	24.0 (14,749)	19.8 (24,586)	21.3 (12,755)
Hypertension	94.8 (58,162)	70.0 (86,832)	62.6 (37,445)
Thromboembolism	4.2 (2,564)	5.1 (6,327)	4.2 (2,491)
Ischemic stroke	0.0 (0)	0.0 (0)	0.0 (0)
Systemic embolism	0.7 (401)	0.9 (1,139)	0.4 (228)
Transient ischemic attack	3.5 (2,175)	4.2 (5,246)	3.8 (2,274)
Vascular disease	17.0 (10,433)	18.9 (23,401)	20.4 (12,187)
Acute myocardial infarction	9.7 (5,981)	12.2 (15,149)	6.8 (4,053)
Coronary procedure	10.4 (6,410)	11.4 (14,189)	16.3 (9,752)
Peripheral artery disease	4.3 (2,630)	3.9 (4,862)	4.1 (2,441)
Concomitant medication a			
Antiplatelet drugs	41.3 (25,362)	52.7 (65,418)	7.3 (4,385)
NSAID	40.8 (25,004)	18.2 (22,534)	26.5 (15,849)
Statins	54.1 (33,218)	35.2 (43,734)	40.6 (24,319)
Modified CHA _2_ DS _2_ -VASc, b mean ± SD	1.9 (0.7)	3.0 (1.3)	3.0 (1.3)
Modified CHA _2_ DS _2_ -VASc, b % ( *n* )			
1	30.0 (18,428)	12.1 (14,995)	13.7 (8,227)
2	45.6 (27,947)	24.1 (29,927)	25.2 (15,078)
≥3	24.4 (14,970)	63.8 (79,198)	61.1 (36,550)
Modified HAS-BLED, c mean ± SD	1.4 (0.9)	2.4 (1.0)	1.6 (0.7)
Modified HAS-BLED, c % ( *n* )			
0	15.1 (9,235)	0.6 (729)	1.2 (741)
1	40.6 (24,888)	19.9 (24,743)	29.9 (17,854)
2	33.1 (20,322)	32.4 (40,169)	45.3 (27,125)
≥3	11.2 (6,900)	47.1 (58,479)	23.6 (14,135)

Abbreviations: IQR, interquartile range; NSAID, nonsteroidal anti-inflammatory drug; OAC, oral anticoagulation; SD, standard deviation; VKA, vitamin K antagonists.

a Concomitant medical therapy within 1 year before baseline.

b Modified CHA _2_ DS _2_ -VASc score included information about medication to define congestive heart failure, hypertension, and diabetes. Information about procedure code was included to define coronary heart disease.

c Modified HAS-BLED score without labile international normalized ratio.

### OAC Initiation

The proportions of patients initiating OACs were 67.9% (67.5–68.3) in Denmark, 67.7% (95% CI: 67.5–68.0) in Sweden, and 69.6% (69.2–70.0) in Finland ( Table 2 ). An expanded time window for the initiation of −180 days to +90 days increased the proportion of initiators slightly to 68.9 (68.5–69.2) in Denmark, 68.7 (68.5–69.0) in Sweden, and 71.1 (70.7–71.4) in Finland. Restriction to patients having a primary diagnosis of AF increased the initiation rates to 75.0 (74.7–75.4) in Denmark and 78.7 (78.4–79.0) in Sweden (data about primary vs. secondary diagnoses of AF were not accessible in Finland due to a previous data management decision).

**Table 2:** Proportion of patients with atrial fibrillation initiating oral anticoagulation (OAC) in Denmark, Sweden, and Finland

	OAC initiation Percentage (95% CI)
Denmark	67.9 (67.5–68.3)
Sweden	67.7 (67.5–68.0)
Finland	69.6 (69.2–70.0)

**Fig. 1:**
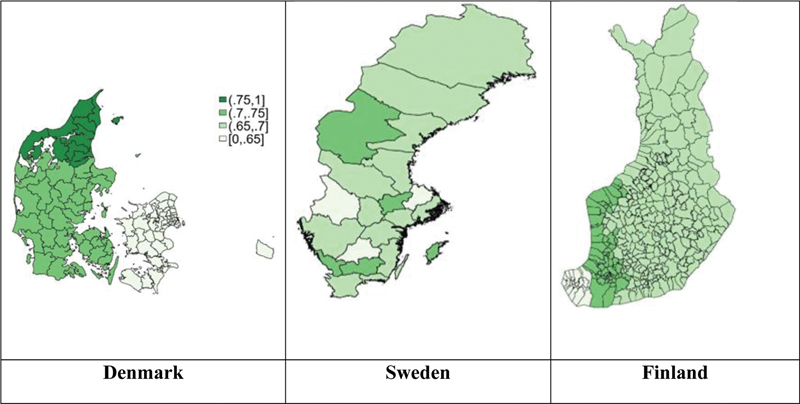
National maps of geographical variation in initiating oral anticoagulation in patients with atrial fibrillation in Denmark, Sweden, and Finland.

**Fig. 2:**
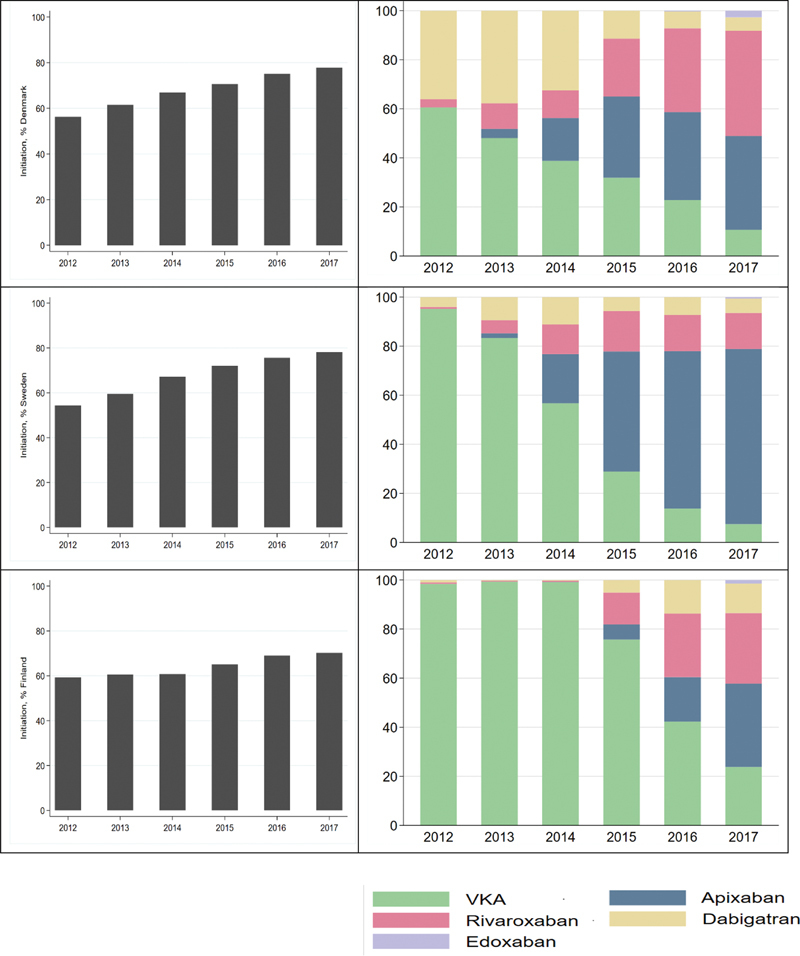
Temporal development of initiating oral anticoagulation in patients with atrial fibrillation (left panel) and temporal development of preferred oral anticoagulant (right panel) in patients with atrial fibrillation in Denmark, Sweden, and Finland.

National maps of the initiation of OAC by administrative regions demonstrated substantial variation within countries, with the most pronounced variation in the initiation of OAC observed in Denmark, ranging from 61 to 77% ( Fig. 1 , Supplementary Tables S9 and S10, available online only ). The overall proportion of patients initiating OAC therapy increased from 56.3 (55.3–57.2) to 77.8 (77.0–78.6) in Denmark and from 54.4 (53.7–55.1) to 78.1 (77.6–78.7) in Sweden, whereas the proportion increased from 59.3 (58.3–60.4) to 70.2 (69.2–71.2) in 2017 in Finland ( Fig. 2 , Supplementary Table S11, available online only ). The transitions from the use of warfarin to DOACs occurred more quickly in Denmark and Sweden than in Finland ( Fig. 2 ). At the end of the study inclusion period in 2017, in Denmark 10.7% of patients were initiated on warfarin, and in Sweden, 7.4% were initiated on warfarin, whereas 23.8% of the patients were initiated on warfarin in Finland ( Fig. 2 , Supplementary Table S12, available online only ).

### Use of Antiplatelet Drugs

The use of antiplatelet drugs decreased substantially over the study period in all countries, from 40.3% (39.3–41.2) in Denmark and 47.6% (46.9–48.3) in Sweden to 20.1% (19.3–20.9) in Denmark and 26.1% (25.5–26.7) in Sweden ( Supplementary Table S13, available online only ). Data are not shown for Finland, because acetylsalicylic acid is very seldom on prescription in Finland.

### Ischemic Stroke

The cumulative incidence (95% CI) of ischemic stroke at 1 year after baseline was highest in Denmark: 2.3% (2.2–2.4) versus 1.9% (1.8–2.0) in both Sweden and Finland ( Table 3 ). In Denmark, the risk of ischemic stroke varied considerably between regions, from 1.7% (1.4–2.0) in the North Denmark region to 2.6% (2.4–3.0) in the capital and 2.7% (2.4–3.0) in the Zealand regions ( Supplementary Table S14 ). In Sweden and Finland, the risk of ischemic stroke varied among regions but with overlapping CIs ( Supplementary Tables S15 and S16 ).

**Table 3:** Cumulative incidences of the outcomes in patients with atrial fibrillation in Denmark, Sweden, and Finland

	Ischemic stroke	Intracerebral hemorrhage	Intracranial bleeding	Other major bleeding	Total mortality
	Percentage (95% CI)				
Denmark	2.3 (2.2–2.4)	0.4 (0.3–0.4)	0.5 (0.4–0.5)	5.4 (5.2–5.6)	11.7 (11.4–12.0)
Sweden	1.9 (1.8–2.0)	0.4 (0.4–0.5)	0.7 (0.6–0.7)	3.3 (3.2–3.4)	10.3 (10.2–10.5)
Finland	1.9 (1.8–2.0)	0.3 (0.3–0.4)	0.5 (0.4–0.5)	3.6 (3.5–3.8)	8.0 (7.7–8.2)

Note: Cumulative incidence of clinical outcomes 1 year after the diagnosis of atrial fibrillation, with death considered a competing event except for total mortality.

### Other Outcomes

The risk of intracerebral hemorrhage did not vary among nations, with a 1-year cumulative incidence of 0.3 to 0.4% ( Table 2 ). Intracranial bleeding was observed more often in Sweden at 0.7% (0.6–0.7) compared with slightly lower bleeding rates of 0.5% (0.4–0.5) in Denmark and Finland. Other major bleeding events were most frequently observed in Denmark. The total mortality was high in all countries, ranging from 8.0 to 11.7% ( Table 3 ). This high mortality corresponded to the study populations consisting of patients with a median age of 73 to 75 years with multiple comorbidities.

### Secular Trend in Outcomes

**Fig. 3:**
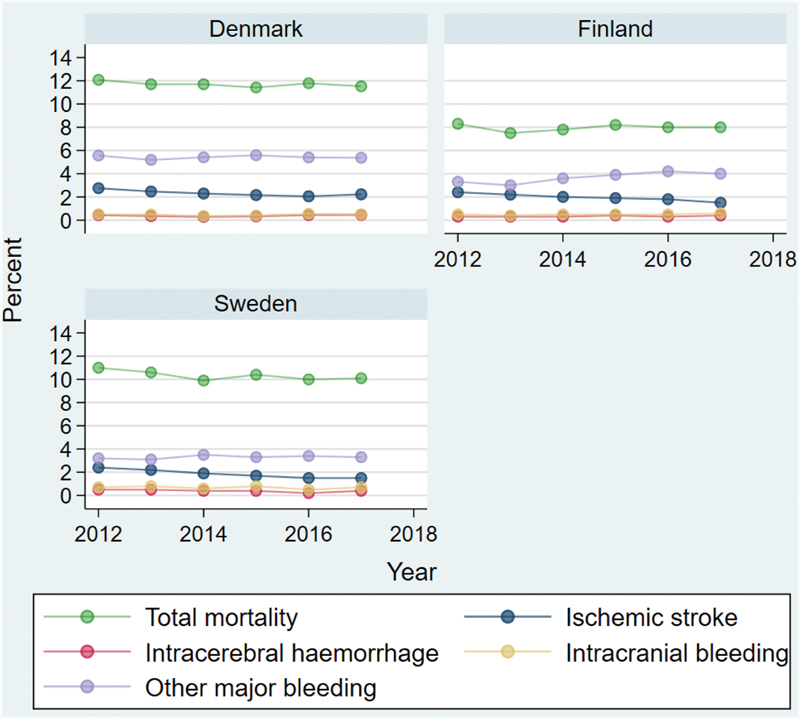
Temporal development of clinical outcomes. Annual cumulative incidences (%) of the outcomes in patients with atrial fibrillation for Denmark, Sweden, and Finland, with death considered a competing event.

The cumulative incidence of ischemic stroke within 1 year after baseline decreased significantly during the study period in all nations: in Denmark, from 2.8% (2.4–3.1) to 2.2% (1.9–2.5); in Sweden, from 2.4% (2.2–2.6) to 1.5% (1.3–1.7); and in Finland, from 2.4% (2.1–2.7) to 1.5% (1.2–1.7) ( Fig. 3 , Table 4 ). This corresponds to an absolute real-world reduction in first-year ischemic stroke risk after AF being diagnosed of 0.6 to 0.9 percentage points and a relative risk reduction of ischemic stroke of 21 to 38%, in parallel with a more frequent use of OAC. The risk of intracerebral and intracranial bleeding did not change throughout the study period.

**Table 4:** Temporal development of clinical outcomes in patients with atrial fibrillation in Denmark, Sweden, and Finland

Year of diagnosis	Ischemic stroke	Intracerebral hemorrhage	Intracranial bleeding	Other major bleeding	All-cause mortality
	Cumulative incidence 1 y after incident atrial fibrillation				
	**Denmark**				
2012	2.8 (2.4–3.1)	0.4 (0.3–0.6)	0.5 (0.4–0.7)	5.6 (5.1–6.0)	12.1 (11.5–12.7)
2013	2.5 (2.2–2.8)	0.3 (0.2–0.5)	0.5 (0.4–0.6)	5.2 (4.8–5.6)	11.7 (11.1–12.3)
2014	2.3 (2.0–2.6)	0.3 (0.2–0.4)	0.4 (0.3–0.5)	5.4 (5.0–5.9)	11.7 (11.1–12.3)
2015	2.2 (1.9–2.5)	0.3 (0.2–0.5)	0.4 (0.3–0.5)	5.6 (5.1–6.0)	11.4 (10.8–12.1)
2016	2.1 (1.8–2.3)	0.4 (0.3–0.6)	0.5 (0.4–0.7)	5.4 (5.0–5.9)	11.8 (11.1–12.4)
2017	2.2 (1.9–2.5)	0.4 (0.3–0.6)	0.5 (0.4–0.7)	5.4 (4.9–5.8)	11.5 (10.9–12.2)
*p-Value for trend*	< 0.001	0.30	0.36	0.18	0.12
	**Sweden**				
2012	2.4 (2.2–2.6)	0.5 (0.4–0.6)	0.7 (0.6–0.9)	3.2 (2.9–3.4)	11.0 (10.6–11.5)
2013	2.2 (2.1–2.4)	0.5 (0.4–0.6)	0.8 (0.7–1.0)	3.1 (2.8–3.3)	10.6 (10.2–11.1)
2014	1.9 (1.7–2.1)	0.4 (0.3–0.5)	0.6 (0.5–0.7)	3.5 (3.2–3.7)	9.9 (9.5–10.3)
2015	1.7 (1.5–1.9)	0.4 (0.4–0.5)	0.8 (0.7–0.9)	3.3 (3.0–3.5)	10.4 (9.9–10.8)
2016	1.5 (1.3–1.7)	0.2 (0.1–0.3)	0.5 (0.4–0.6)	3.4 (3.1–3.6)	10.0 (9.6–10.5)
2017	1.5 (1.3–1.7)	0.4 (0.3–0.5)	0.7 (0.6–0.8)	3.3 (3.1–3.6)	10.1 (9.7–10.5)
*p-Value for trend*	< 0.001	0.01	0.02	0.18	< 0.001
	**Finland**				
2012	2.4 (2.1–2.7)	0.3 (0.3–0.4)	0.5 (0.4–0.6)	3.3 (3.0–3.7)	8.3 (7.8–8.9)
2013	2.2 (1.9–2.4)	0.3 (0.2–0.4)	0.4 (0.3–0.5)	3.0 (2.7–3.3)	7.5 (7.0–8.0)
2014	2.0 (1.7–2.3)	0.3 (0.2–0.5)	0.5 (0.4–0.7)	3.6 (3.3–4.0)	7.8 (7.3–8.3)
2015	1.9 (1.6–2.1)	0.4 (0.3–0.5)	0.5 (0.4–0.7)	3.9 (3.6–4.3)	8.2 (7.7–8.8)
2016	1.8 (1.6–2.0)	0.3 (0.2–0.5)	0.5 (0.3–0.6)	4.2 (3.8–4.6)	8.0 (7.5–8.6)
2017	1.5 (1.2–1.7)	0.4 (0.3–0.5)	0.6 (0.4–0.7)	4.0 (3.6–4.4)	8.0 (7.5–8.6)
*p-Value for trend*	< 0.001	0.06	0.10	< 0.001	0.001

Note: Cumulative incidences 1 year after incident atrial fibrillation of the outcomes for the three nations, with death considered a competing event.

Other major bleeds were most frequently seen in Denmark, and a small increase in other major bleeds was observed in Finland ( Fig. 3 , Table 4 ).

## Discussion

In this multinational cohort study of OAC-naive patients with incident AF, we found substantial variations in OAC initiation and the 1-year risk of ischemic strokes across and within Denmark, Finland, and Sweden. Within-nation variation was most pronounced in Denmark. Furthermore, our data documented differences in the speed of transition of OAC preferences from warfarin to DOACs.

Variations in OAC use among four international regions, North America, Europe, Asia, and Latin America, has recently been reported. 18 Reasons for geographical variations in the use of OACs have been suggested to stem from variations in local AF guidelines, variations in health care systems, or socioeconomic factors. 18

In Denmark, there was no regulatory preference for DOACs over vitamin K antagonist (VKA) during the first few years after the introduction of DOACs, but since 2016, DOACs have been preferred over VKA by regulatory authorities in Denmark. In Finland, a slower transition from VKA to DOACs can be explained by a lower reimbursement rate for DOACs from 2013 to 2015. 19

The 1-year risk of ischemic stroke decreased in all countries over the study period, whereas the intracerebral bleeding risk remained unchanged. A correlation between a higher uptake of OACs and a reduced risk of stroke has also been reported in England, 10 Germany, 7 Sweden, 6 and Italy. 9 The finding of a non-increase in the risk of intracerebral and intracranial bleeds despite a higher user rate of OAC can be explained by a lower use of antiplatelet drugs in combination with a lower risk of intracerebral and intracranial bleeds associated with the use of DOACs compared with warfarin. 2

We observed a gradual increase in the use of OACs by patients with AF. The explanation for this phenomenon is highly complex. It may include approval by the EMA of dabigatran and rivaroxaban for the prevention of ischemic stroke and systemic embolism in AF in 2011, apixaban in 2012, and edoxaban in 2015 in combination with the gradual evolution and implementation of ESC AF guidelines and a decreasing reluctance among clinicians to use OACs.

At the end of our study period in 2017, 78% of patients with AF and CHA _2_ DS _2_ -VASc scores ≥1 in men and ≥2 in women initiated OAC therapy, predominantly DOACs. This is consistent with a recent systematic review and meta-analysis of worldwide trends in OAC use among patients with AF from 2010 to 2018. 3

Comprehensive management of patients with AF adhering to the “Atrial Fibrillation Better Care” pathway has demonstrated promising results by lowering the risk of all-cause death, cardiovascular death, and major bleeding. 20 A global implementation and delivery of structured care of patients with AF can also reduce variations in treatments and outcomes.

## Strengths and Limitations

The novelty of the present study derives from the presentation of data on both OAC use and a range of clinical outcomes, including ischemic stroke and specified bleeding outcomes, in multiple nationwide population-based cohorts with accurate follow-up information. Our results must be interpreted bearing in mind the limitations of this study, mainly the challenges inherent to the retrospective study design and the use of administrative data. Thus, our findings represent associations and not necessarily causality. Moreover, information bias may be present due to inaccurately recorded data in the health care registries.

It can be challenging to determine whether an ischemic stroke is a recurrent stroke based on information retrieved from administrative registries because a stroke patient can be transferred between hospital departments and hospitals, and each contact will be assigned a stroke diagnosis. Therefore, we excluded patients with a history of stroke to increase the validity of our study. A diagnosis of ischemic stroke reported to Scandinavian patient registries has a moderate to high positive predictive value. 21 22 23 The positive predictive value of a diagnosis of a transient ischemic attack recorded in administrative registries has a low-to-moderate positive predictive value 24 and was therefore not included in our study. Differences in coding practice across countries may exist. We had no information on whether the patients used the prescribed medicine. We adjusted for several relevant covariates but could not rule out residual confounding from unmeasured variables, including disease severity, socioeconomic factors, and patient preferences. The organization and structure of the health care systems in the Nordic countries are similar and characterized by free access to tax-financed hospital care, but the results may not be generalizable to other health care systems.

## Conclusion

We documented variations in transition rates of preferences for DOACs over warfarin, increasing proportions of patients initiating OAC, geographical variations in the proportion of patients initiating OAC, and variations in clinical outcomes among patients with AF in Denmark, Sweden, and Finland. The secular risk of ischemic stroke decreased in all countries. International and intraregional variations in the initiation of OAC therapy, as well as in clinical outcomes, were observed despite comparable health care systems. Global implementation of structured care of patients with AF could reduce future variations in the treatment and outcomes of AF.
