# Supplementary material for: Geographical Variation in the Use of Oral Anticoagulation and Clinical Outcomes among Patients with Atrial Fibrillation in Denmark, Sweden, and Finland
Source: TH Open. 2023 Jun 6;7(2):e133–42. doi: 10.1055/a-2080-6171 (PMC10243918; doi:10.1055/a-2080-6171)
Supplement: Supplementary file 1 — Supplementary Material [file 10-1055-a-2080-6171-s23020008.pdf]

Supplementary Materials

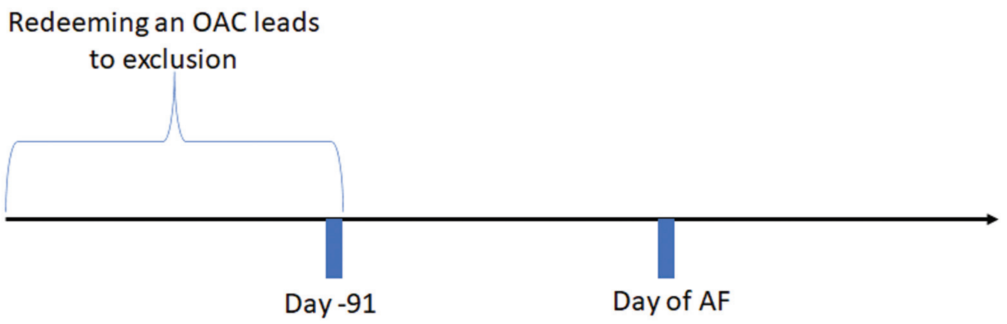

Supplementary Fig. S1 Time window of oral anticoagulation and exclusion.

Supplementary Table S1 Definition of valvular AF

Exclusion criteria	Classification system	Diagnosis or procedure code	Period
Mitral stenosis, or	ICD-10<br>(A or B diagnosis)	DI050, DI052, DI342	Before or on the day of the AF diagnosis
	NCSP	KFKA	
Mechanical prosthetic heart valves	NCSP	KFCA60, KFGE00, KFJF00, KFKD00, KFMD00	Before or on the day of the AF diagnosis

Abbreviations: AF, atrial fibrillation; NCSP, the Nordic Medical Statistics Committees Classification of Surgical Procedures.

 © 2023. The Author(s).

Supplementary Table S2 Definition of the modified CHA_2DS_2-VASc score

Risk factor	Definition	ICD-10	ATC codes	Procedure code (A)
Congestive heart failure	Defined from at least one diagnosis in combination with at least one prescription of loop diuretics (prescribed within 1 year before the AF diagnosis)[1]	I11.0; I13.0; I13.2; I42.0; I50	C03C	
Hypertension	Defined from diagnosis AND/OR treatment with at least two classes of antihypertensive drugs (both drugs prescribed within 1 year before the AF diagnosis)[2]	I10; I11; I12; I13; I15	Classes of antihypertensive drugs:<br>α-Adrenergic blockers: C02A; C02B; C02C<br>Non-loop diuretics: C02DA; C02L; C03A; C03B; C03D; C03E; C03X; C07B; C07C; C07D; C08G; C09BA; C09DA; C09XA52<br>Vasodilators: C02DB; C02DD; C02DG; C04; C05<br>β-Blockers: C07<br>Calcium channel blockers: C08; C09BB; C09DB<br>Renin–angiotensin system inhibitors: C09	
Diabetes mellitus	Defined from diagnosis AND/OR treatment with blood glucose lowering drugs	E10; E11; E12; E13; E14; H360	A10A; A10B	
Previous thromboembolism	Defined as ischemic stroke AND/OR transient ischemic attack (TIA) AND/OR systemic embolism			
- Ischemic stroke	Defined from the diagnosis of ischemic stroke and unspecified stroke. The exception of DI636 is due to the venous origin	I63 (except I636); DI64		
- TIA	Defined from diagnosis	G45 (except G453 and G454)		
- Systemic embolism	Defined from the diagnosis of embolus and thrombosis in arteries, acute vascular disorders of intestine and ischemia and infarction of kidney	I74; K550; N280		
Vascular disease	Defined as MI AND/OR PAD AND/OR coronary procedure			
- Myocardial infarction	Defined from diagnosis	I21; I22		
- Peripheral artery disease	Defined from diagnosis	I65; I70		
- Coronary procedures	Defined from code of operation			KFN

 © 2023. The Author(s).

Supplementary Table S3 Definition of the clinical outcomes

Outcome	Definition	ICD-10
Ischemic stroke	Defined from the diagnosis of ischemic stroke and unspecified stroke. The exception of DI636 is due to the venous origin	I63 (except DI636); I64
Intracerebral hemorrhage	Defined from diagnosis of intracerebral hemorrhage	I61
Intracranial bleeding	Defined from diagnosis of subarachnoid hemorrhage, intracerebral hemorrhage, and other nontraumatic intracranial hemorrhage	I60; I61; I62
Other major bleeding	Defined from any of the diagnosis below (as in HAS-BLED excluding diagnosis of other nontraumatic intracranial hemorrhage)	
	Anemia caused by bleeding	D500; D62
	Gastrointestinal bleeding defined from:	
	Oesophageal and gastric varices with bleeding	I850; I864A
	Bleeding peptic ulcer	K250; K252; K254; K256; K260; K262; K264; K266; K270; K272; K274; K276; K280; K282; K284; K286
	Other gastrointestinal bleeding	K290; K298A; K625; K920; K921; K922
	Hemothorax and respiratory tract bleeding	J942; R04
	Hematuria	N02; R31
	Unspecified bleeding	R58

Abbreviation: NCSP, the Nordic Medical Statistics Committees Classification of Surgical Procedures.

Supplementary Table S4 Definition of OAC therapy

Medication	ATC code
VKA	B01AA
Warfarin	B01AA03
Phenprocoumon	B01AA04
NOAC	
Rivaroxaban	B01AF01, B01AX06
Apixaban	B01AF02
Dabigatran	B01AE07
Edoxaban	B01AF03

Abbreviations: NOAC, non-vitamin K antagonist oral anticoagulant; OAC, oral anticoagulation; VKA, vitamin K antagonist.

 © 2023. The Author(s).

Supplementary Table S5 Definition of the modified HAS-BLED score

Risk factor	Definition	ICD-10	ATC codes
Hypertension	The same as for CHA_2DS_2-VASc		
Abnormal renal function	Defined from diagnosis	E102; E112; E132; E142; I12; I13; N03; N04; N05; N07; N08; N11; N14; N158; N159; N16; N18 (except DN181 and N182); N19; N26; Q61	
Abnormal liver function	Defined from diagnosis	B18; C22; D684C; K70; K71 (except K710; K711A; K711B; K712 and K716); K72; K73; K74; K75; K76; K77; Q618A; Z944	
Stroke	Defined from diagnosis of subarachnoid hemorrhage, intracerebral hemorrhage, cerebral infarction, and unspecified stroke	I60; I61; I63; I64	
Bleeding	Defined from any of the diagnosis below		
	Anemia caused by bleeding	D500; D62	
	Other nontraumatic intracranial hemorrhage	I62	
	Gastrointestinal bleeding defined from any of the following:		
	Oesophageal and gastric varices with bleeding	I850; I864A	
	Bleeding peptic ulcer	K250; K252; K254; K256; K260; K262; K264; K266; K270; K272; K274; K276; K280; K282; K284; K286	
	Other gastrointestinal bleeding	K290; K298A; K625; K920; K921; K922	
	Hemothorax and respiratory tract bleeding	J942; R04	
	Hematuria	N02; R31	
	Unspecified bleeding	R58	
Antiplatelet drugs	Defined from treatment with antiplatelet drugs *or* NSAID		B01AC06; B01AC04; B01AC22; B01AC24; B01AC07; B01AC30; M01A; N02BA01
Alcohol-related disease	Defined from alcohol-related diagnosis and adverse alcohol consumption reported during hospitalization	E244; E512; E52; F10; G312; G621; G721; I426; K292; K70; K860; L278A; R780; T51; Z502; Z714; Z721; K852; T500A	

Abbreviation: NSAID, nonsteroidal anti-inflammatory drug.

 © 2023. The Author(s).

Supplementary Table S6 Definitions of concomitant medication

Medication	ATC code
Antiplatelet drugs	
Acetylsalicylic acid	B01AC06, N02BA01
Clopidogrel	B01AC04
Prasugrel	B01AC22
Ticagrelor	B01AC24
Dipyridamole	B01AC07
Combination: acetylsalicylic acid, dipyridamole	B01AC30
NSAID	M01A
Statins	C10AA, C10BA

Abbreviation: NSAID, nonsteroidal anti-inflammatory drug.

Supplementary Table S7 Overview of adjusted regression analyses

Outcome	Model 1	Model 2
Initiation	Age (linear), sex	Age (linear), sex, modified CHA_2DS_2-VASc (factor), modified HAS-BLED (factor), statins, and antiplatelets
Ischemic stroke	Age (linear), sex	Age (linear), sex, the components of modified CHA_2DS_2-VASc, statins, antiplatelet drugs, and NSAID
Intracerebral hemorrhage	Age (linear), sex	Age (linear), sex, and components of the modified HAS-BLED
Intracranial bleeding	Age (linear), sex	Age (linear), sex, and components of the modified HAS-BLED
Other major bleeding	Age (linear), sex	Age (linear), sex, and components of the modified HAS-BLED
Mortality	Age (linear), sex	Age (linear), sex, modified CHA_2DS_2-VASc (factor), modified HAS-BLED (factor), statins, and antiplatelet

Supplementary Table S8 Flowchart of study cohorts in Denmark, Sweden, and Finland

	Denmark	Sweden	Finland
	Number of individuals included in study cohorts		
Patients with incident atrial fibrillation diagnosis	474,459	404,423	120,938
Exclude for atrial fibrillation before 2012 and after 2017	119,271	215,068	109,850
Exclude for age < 40 and age ≥ 90 y	108,530	190,583	100,011
Exclude for civil registration status as inactive or disappeared	108,180	NA[a]	NA[a]
Exclude for history of valvular atrial fibrillation	107,453	188,997	99,421
Exclude for history of stroke	93,642	169,315	92,136
Exclude for immigration < 5 y before baseline	93,454	167,002	NA
Exclude for redeemed prescription of oral anticoagulant > 90 d before baseline	77,662	140,231	70,728
Exclude for a modified CHA_2DS_2-VASc score of 0 in men and 1 in women	61,345	124,120	59,855
Study cohorts	61,345	124,120	59,855

[a]Codes for civil registration status as inactive or disappeared are not available in Sweden and Finland.

 © 2023. The Author(s).

Supplementary Table S9 Percentage of patients with atrial fibrillation initiating oral anticoagulation by region in Denmark, Sweden, and Finland

	Percentage (95% CI) of patients initiating oral anticoagulation
**Danish regions**	
Capital	61.5 (60.8–62.2)
North	77.0 (76.0–78.0)
Central	72.4 (71.6–73.1)
South	71.7 (71.0–72.5)
Zealand	61.3 (60.3–62.2)
**Swedish regions**	
Stockholms län	65.9 (65.3–66.6)
Uppsala län	65.0 (63.5–66.5)
Södermanlands län	65.8 (64.3–67.3)
Östergötlands län	69.0 (67.8–70.2)
Jönköpings län	64.8 (63.5–66.2)
Kronobergs län	74.4 (72.6–76.1)
Kalmar län	68.5 (67.0–70.0)
Gotlands län	72.0 (69.1–74.8)
Blekinge län	69.1 (67.2–71.0)
Skåne län	67.1 (66.4–67.8)
Hallands län	73.7 (72.5–75.0)
Västra Götalands län	68.1 (67.5–68.7)
Värmlands län	64.7 (63.3–66.2)
Örebro län	65.9 (64.3–67.4)
Västmanlands län	72.9 (71.5–74.4)
Dalarnas län	70.0 (68.6–71.4)
Gävleborgs län	69.5 (68.2–70.8)
Västernorrlands län	65.4 (63.9–66.9)
Jämtlands län	72.4 (70.2–74.5)
Västerbottens län	67.5 (66.0–69.1)
Norrbottens län	67.1 (65.7–68.6)
**Finish regions (university hospital regions)**	
Tampere Region	70.2 (69.3–71.1)
Helsinki Region	68.1 (67.5–68.7)
Kuopio Region	68.2 (67.2–69.1)
Turku Region	74.5 (73.6–75.3)
Oulu Region	69.9 (68.9–70.9)

Supplementary Table S10 Adjusted relative risks for initiation of oral anticoagulation in patients with atrial fibrillation by region in Denmark, Sweden, and Finland

	Model 1 Relative risk (95% CI)	Model 2 Relative risk (95% CI)
**Danish regions**		
Capital	1	1
North	1.24 (1.23–1.27)	1.24 (1.21–1.26)
Central	1.17 (1.15–1.19)	1.17 (1.15–1.18)
South	1.15 (1.14–1.18)	1.15 (1.14–1.17)
Zealand	0.99 (0.98–1.01)	1.00 (0.98–1.02)
**Swedish regions**		
Stockholms län	1	1
Uppsala län	0.99 (0.96–1.01)	0.97 (0.95–1.00)
Södermanlands län	1.00 (0.98–1.02)	0.98 (0.96–1.00)
Östergötlands län	1.05 (1.03–1.07)	1.04 (1.02–1.06)
Jönköpings län	0.99 (0.96–1.01)	0.99 (0.97–1.02)
Kronobergs län	1.13 (1.10–1.16)	1.11 (1.08–1.14)
Kalmar län	1.04 (1.02–1.07)	1.03 (1.01–1.06)
Gotlands län	1.09 (1.05–1.14)	1.09 (1.05–1.13)
Blekinge län	1.05 (1.02–1.08)	1.04 (1.01–1.07)
Skåne län	1.02 (1.01–1.03)	1.01 (1.00–1.02)
Hallands län	1.12 (1.10–1.14)	1.12 (1.09–1.14)
Västra Götalands län	1.03 (1.02–1.05)	1.02 (1.00–1.03)
Värmlands län	0.98 (0.96–1.01)	0.97 (0.94–0.99)
Örebro län	1.00 (0.97–1.03)	0.98 (0.95–1.00)
Västmanlands län	1.11 (1.08–1.13)	1.09 (1.07–1.12)
Dalarnas län	1.06 (1.04–1.09)	1.05 (1.03–1.07)
Gävleborgs län	1.06 (1.03–1.08)	1.03 (1.01–1.06)
Västernorrlands län	0.99 (0.97–1.02)	0.98 (0.95–1.00)
Jämtlands län	1.10 (1.07–1.13)	1.09 (1.05–1.12)
Västerbottens län	1.03 (1.00–1.05)	1.01 (0.98–1.03)
Norrbottens län	1.02 (1.00–1.04)	1.00 (0.98–1.03)
**Finish regions (university hospital regions)**		
Tampere Region	1	1
Helsinki Region	0.97 (0.96–0.99)	0.97 (0.96–0.99)
Kuopio Region	0.97 (0.95–0.99)	0.97 (0.95–0.99)
Turku Region	1.06 (1.04–1.08)	1.05 (1.04–1.07)
Oulu Region	1.00 (0.98–1.02)	0.99 (0.97–1.01)

Notes: Model 1—Adjusted for age (linear) and sex. Model 2—Adjusted for age (linear), sex, CHA_2DS_2-VASc (factor), HAS-BLED (factor), statins, and antiplatelets.

 © 2023. The Author(s).

Supplementary Table S11 Temporal development in initiation of oral anticoagulation in patients with atrial fibrillation in Denmark, Sweden, and Finland

Year of diagnosis	Denmark	Sweden	Finland
	Percentage (95% CI)		
2012	56.3 (55.3–57.2)	54.4 (53.7–55.1)	59.3 (58.3–60.4)
2013	61.5 (60.6–62.5)	59.5 (58.8–60.2)	60.6 (59.6–61.7)
2014	66.9 (65.9–67.8)	67.2 (66.6–67.9)	60.7 (59.6–61.7)
2015	70.6 (69.8–71.5)	72.0 (71.4–72.6)	65.1 (64.1–66.2)
2016	75.1 (74.2–75.9)	75.6 (75.0–76.2)	69.0 (68.0–70.0)
2017	77.8 (77.0–78.6)	78.1 (77.6–78.7)	70.2 (69.2–71.2)

Supplementary Table S12 Temporal development in use of specific oral anticoagulants in patients with atrial fibrillation in Denmark, Sweden, and Finland

Year of diagnosis	Oral anticoagulant	Denmark	Sweden	Finland
		Percentage (95% CI)		
2012	Warfarin	60.6 (46.9–49.3)	95.2 (95.1–95.3)	98.4 (98.3–98.5)
	Dabigatran	36.0 (34.8–37.3)	4.0 (3.9–4.1)	1.0 (0.9–1.1)
	Rivaroxaban	3.3 (2.9–3.8)	0.7 (0.7–0.8)	0.5 (0.4–0.6)
	Apixaban	0	0	0
	Edoxaban	0	0	0
2013	Warfarin	48.1 (46.9–49.3)	83.3 (83.1–83.5)	99.4 (99.3–99.5)
	Dabigatran	37.7 (36.6–38.9)	9.4 (9.2–9.6)	0.2 (0.2–0.2)
	Rivaroxaban	10.4 (9.7–11.2)	5.4 (5.3–5.5)	0.4 (0.4–0.5)
	Apixaban	3.8 (3.3–4.2)	1.9 (1.8–2.0)	0
	Edoxaban	0	0	0
2014	Warfarin	38.8 (37.7–40.0)	56.7 (56.4–57.0)	99.1 (99.0–99.2)
	Dabigatran	32.4 (31.3–33.6)	11.1 (10.9–11.3)	0.3 (0.3–0.4)
	Rivaroxaban	11.3 (10.6–12.1)	12.1 (11.9–12.3)	0.5 (0.4–0.6)
	Apixaban	17.4 (16.6–18.4)	20.1 (19.9–20.3)	0.1 (0.1–0.1)
	Edoxaban	0	0	0
2015	Warfarin	31.9 (30.8–33.0)	28.8 (28.6–29.1)	75.7 (75.4–76.0)
	Dabigatran	11.4 (10.7–12.1)	5.7 (5.6–5.8)	5.1 (4.9–5.3)
	Rivaroxaban	23.5 (22.6–24.5)	16.5 (16.3–16.7)	12.9 (12.6–13.2)
	Apixaban	33.2 (32.1–34.3)	49.0 (48.7–49.3)	6.2 (6.0–6.4)
	Edoxaban	0	0	0
2016	Warfarin	22.7 (21.8–23.7)	13.8 (13.6–14.0)	42.3 (41.9–42.7)
	Dabigatran	6.8 (6.3–7.4)	7.3 (7.2–7.5)	13.7 (13.4–14.0)
	Rivaroxaban	34.2 (33.1–35.3)	14.8 (14.6–15.0)	25.9 (25.6–26.3)
	Apixaban	35.9 (34.8–37.0)	64.2 (63.9–64.5)	18.1 (17.8–18.4)
	Edoxaban	0.4 (0.24–0.52)	0.0	0.0
2017	Warfarin	10.7 (10.0–11.4)	7.4 (7.3–7.6)	23.8 (23.5–24.1)
	Dabigatran	5.5 (5.0–6.0)	5.9 (5.8–6.0)	12.1 (11.8–12.4)
	Rivaroxaban	42.9 (41.8–44.0)	14.7 (14.5–14.9)	28.8 (28.4–29.2)
	Apixaban	38.2 (37.1–39.3)	71.4 (71.2–71.7)	34.0 (33.6–34.4)
	Edoxaban	2.7 (2.35–3.08)	0.6 (0.6–0.6)	1.4 (1.3–1.5)

 © 2023. The Author(s).

Supplementary Table S13 Temporal development in initiation[a] of antiplatelet drugs in patients with atrial fibrillation in Denmark, Sweden, and Finland

Year of diagnosis	Denmark	Sweden	Finland[b]
	Percentage (95% CI)		
2012	40.3 (39.3–41.2)	47.6 (46.9–48.3)	5.0 (4.6–5.4)
2013	33.0 (32.1–33.9)	40.3 (39.6–40.9)	5.0 (4.6–5.4)
2014	29.0 (28.1–29.9)	33.2 (32.6–33.9)	4.4 (4.0–4.8)
2015	24.8 (24.0–25.7)	29.6 (28.9–30.2)	4.4 (4.0–4.8)
2016	21.9 (21.1–22.7)	27.3 (26.7–27.9)	3.9 (3.5–4.3)
2017	20.1 (19.3–20.9)	26.1 (25.5–26.7)	3.7 (3.3–4.0)

[a]Initiation within 1 year after diagnosis of atrial fibrillation.
[b]Acetylsalicylic acid is very seldom on prescription in Finland.

 © 2023. The Author(s).

Supplementary Table S14 Cumulative incidences of the outcomes in patients with atrial fibrillation 1 year after baseline by region in Denmark, Sweden, and Finland

	Ischemic stroke	Intracerebral hemorrhage	Intracranial bleeding	Other major bleeding	Total mortality
	Percentage (95% CI)				
	**Danish regions**				
Capital	2.6 (2.4–2.9)	0.3 (0.3–0.4)	0.4 (0.3–0.5)	5.0 (4.7–5.3)	11.4 (10.9–11.9)
North	1.7 (1.4–2.0)	0.3 (0.2–0.5)	0.5 (0.3–0.6)	5.1 (4.5–5.6)	11.0 (10.2–11.7)
Central	1.9 (1.7–2.2)	0.4 (0.3–0.6)	0.5 (0.4–0.6)	5.2 (4.9–5.6)	10.0 (9.5–10.5)
South	1.9 (1.7–2.2)	0.3 (0.2–0.4)	0.4 (0.3–0.5)	5.1 (4.8–5.5)	10.3 (9.8–10.8)
Zealand	2.7 (2.4–3.0)	0.3 (0.2–0.4)	0.4 (0.3–0.5)	5.3 (4.9–5.8)	13.7 (13.0–14.4)
	**Swedish regions**				
Stockholms län	1.8 (1.6–2.0)	0.4 (0.3–0.5)	0.8 (0.6–0.9)	3.5 (3.3–3.8)	9.8 (9.4–10.2)
Uppsala län	1.7 (1.2–2.1)	0.4 (0.2–0.6)	0.5 (0.3–0.8)	2.6 (2.0–3.2)	9.4 (8.4–10.4)
Södermanlands län	1.9 (1.5–2.4)	0.3 (0.0–0.5)	0.5 (0.2–0.8)	3.1 (2.6–3.7)	10.3 (9.3–11.3)
Östergötlands län	1.7 (1.4–2.1)	0.5 (0.3–0.7)	0.8 (0.6–1.0)	3.0 (2.5–3.5)	10.4 (9.6–11.2)
Jönköpings län	2.0 (1.6–2.4)	0.4 (0.2–0.6)	0.8 (0.5–1.0)	3.3 (2.7–3.8)	11.4 (10.5–12.3)
Kronobergs län	1.6 (1.0–2.1)	0.3 (0.0–0.5)	0.5 (0.2–0.9)	3.4 (2.7–4.1)	10.3 (9.0–11.5)
Kalmar län	1.8 (1.3–2.2)	0.4 (0.2–0.6)	0.5 (0.3–0.8)	3.6 (3.0–4.2)	11.8 (10.8–12.8)
Gotlands län	1.4 (0.5–2.3)	0.3 (0.0–0.8)	0.9 (0.4–1.5)	3.4 (2.2–4.5)	9.8 (7.8–11.8)
Blekinge län	2.0 (1.4–2.5)	0.6 (0.3–0.8)	0.8 (0.5–1.2)	2.8 (2.0–3.5)	10.9 (9.6–12.2)
Skåne län	1.8 (1.5–2.0)	0.4 (0.3–0.5)	0.6 (0.5–0.7)	3.4 (3.1–3.6)	10.5 (10.0–11.0)
Hallands län	1.4 (1.0–1.8)	0.4 (0.2–0.6)	0.5 (0.2–0.7)	4.2 (3.7–4.8)	10.2 (9.3–11.2)
Västra Götalands län	1.8 (1.6–2.0)	0.4 (0.3–0.5)	0.6 (0.5–0.7)	2.9 (2.6–3.1)	10.0 (9.6–10.4)
Värmlands län	2.3 (1.9–2.8)	0.4 (0.2–0.6)	0.7 (0.5–1.0)	4.1 (3.6–4.7)	11.8 (10.8–12.7)
Örebro län	2.1 (1.6–2.6)	0.5 (0.3–0.7)	0.8 (0.5–1.0)	3.0 (2.4–3.6)	10.9 (9.9–12.0)
Västmanlands län	2.3 (1.8–2.8)	0.5 (0.3–0.7)	0.9 (0.6–1.2)	3.5 (2.9–4.1)	11.2 (10.1–12.3)
Dalarnas län	2.0 (1.6–2.5)	0.5 (0.3–0.7)	0.7 (0.5–1.0)	3.4 (2.9–4.0)	10.1 (9.1–11.1)
Gävleborgs län	2.1 (1.7–2.5)	0.5 (0.3–0.7)	0.8 (0.5–1.0)	3.5 (2.9–4.0)	10.4 (9.5–11.3)
Västernorrlands län	2.0 (1.6–2.5)	0.4 (0.2–0.6)	0.5 (0.3–0.8)	2.9 (2.3–3.5)	11.2 (10.2–12.2)
Jämtlands län	2.1 (1.4–2.8)	0.5 (0.2–0.9)	0.9 (0.5–1.3)	2.3 (1.4–3.1)	9.5 (8.0–11.0)
Västerbottens län	2.1 (1.7–2.6)	0.4 (0.1–0.6)	0.9 (0.6–1.2)	3.1 (2.5–3.7)	10.0 (9.0–11.0)
Norrbottens län	2.4 (1.9–2.8)	0.4 (0.2–0.6)	0.7 (0.5–1.0)	3.4 (2.8–4.0)	10.2 (9.2–11.1)
	**Finish regions (university hospital regions)**				
Tampere Region	2.0 (1.7–2.3)	0.4 (0.3–0.5)	0.5 (0.4–0.6)	0.4 (0.3–0.4)	8.1 (7.6–8.7)
Helsinki Region	2.0 (1.8–2.2)	0.3 (0.2–0.4)	0.4 (0.3–0.5)	0.4 (0.3–0.4)	7.9 (7.6–8.3)
Kuopio Region	2.1 (1.8–2.4)	0.4 (0.3–0.6)	0.6 (0.4–0.7)	0.4 (0.3–0.4)	7.7 (7.2 -8.2)
Turku Region	1.8 (1.5–2.0)	0.4 (0.3–0.5)	0.5 (0.4–0.7)	0.4 (0.3–0.4)	8.2 (7.7–8.7)
Oulu Region	1.9 (1.6–2.3)	0.4 (0.3–0.6)	0.5 (0.4–0.7)	0.4 (0.3–0.4)	8.0 (7.5–8.7)

 © 2023. The Author(s).

Supplementary Table S15 Adjusted relative risks (95% CI) 1 year after baseline for the clinical outcomes in patients with atrial fibrillation by region in Denmark, Sweden, and Finland: adjusted as Model 1

	Ischemic stroke	Intracerebral hemorrhage	Intracranial bleeding	Other major bleeding	Total mortality
	Relative risk (95% CI)				
	Danish regions				
Capital	1	1	1	1	1
North	0.65 (0.53–0.80)	1.04 (0.61–1.78)	1.17 (0.75–1.81)	0.99 (0.87–1.12)	0.97 (0.89–1.05)
Central	0.75 (0.64–0.88)	1.43 (0.95–2.15)	1.30 (0.91–1.86)	1.02 (0.92–1.13)	0.91 (0.85–0.98)
South	0.75 (0.64–0.87)	1.03 (0.68–1.56)	0.96 (0.66–1.40)	0.97 (0.87–1.07)	0.90 (0.84–0.97)
Zealand	1.07 (0.92–1.24)	0.96 (0.60–1.52)	0.90 (0.60–1.37)	1.07 (0.96–1.19)	1.13 (1.05–1.21)
	Swedish regions				
Stockholms län	1	1	1	1	1
Uppsala län	0.87 (0.67–1.13)	1.10 (0.64–1.91)	0.77 (0.49–1.22)	0.75 (0.60–0.92)	0.98 (0.90–1.08)
Södermanlands län	1.03 (0.83–1.29)	0.68 (0.29–1.56)	0.66 (0.39–1.11)	0.83 (0.68–1.00)	1.07 (0.98–1.17)
Östergötlands län	1.01 (0.83–1.21)	1.43 (0.98–2.10)	1.08 (0.82–1.43)	0.82 (0.70–0.97)	1.04 (0.97–1.12)
Jönköpings län	1.03 (0.84–1.25)	1.01 (0.60–1.71)	0.96 (0.69–1.34)	0.89 (0.76–1.05)	1.13 (1.05–1.22)
Kronobergs län	0.73 (0.51–1.04)	0.64 (0.22–1.85)	0.73 (0.41–1.28)	0.97 (0.79–1.18)	1.04 (0.94–1.16)
Kalmar län	0.92 (0.73–1.17)	0.84 (0.43–1.64)	0.65 (0.39–1.09)	0.97 (0.82–1.14)	1.14 (1.05–1.24)
Gotlands län	0.69 (0.37–1.29)	1.11 (0.40–3.07)	1.39 (0.84–2.28)	0.88 (0.62–1.24)	0.96 (0.79–1.15)
Blekinge län	1.07 (0.82–1.38)	1.71 (1.08–2.72)	1.18 (0.80–1.72)	0.78 (0.61–1.01)	1.07 (0.97–1.19)
Skåne län	0.93 (0.81–1.07)	1.12 (0.81–1.55)	0.79 (0.62–1.00)	0.91 (0.83–1.01)	1.08 (1.03–1.14)
Hallands län	0.75 (0.58–0.98)	0.93 (0.52–1.66)	0.56 (0.32–0.97)	1.12 (0.97–1.28)	1.08 (1.00–1.17)
Västra Götalands län	0.96 (0.85–1.09)	1.12 (0.82–1.53)	0.77 (0.61–0.97)	0.75 (0.68–0.84)	1.00 (0.95–1.05)
Värmlands län	1.16 (0.97–1.40)	1.29 (0.82–2.03)	1.03 (0.74–1.42)	1.08 (0.94–1.25)	1.20 (1.12–1.30)
Örebro län	1.22 (1.00–1.49)	1.45 (0.93–2.28)	1.01 (0.70–1.46)	0.86 (0.71–1.04)	1.10 (1.01–1.21)
Västmanlands län	1.24 (1.02–1.51)	1.21 (0.72–2.04)	1.07 (0.75–1.52)	0.95 (0.80–1.14)	1.14 (1.05–1.24)
Dalarnas län	1.04 (0.84–1.29)	1.35 (0.85–2.12)	0.96 (0.67–1.38)	0.93 (0.79–1.11)	1.05 (0.96–1.15)
Gävleborgs län	1.10 (0.90–1.33)	1.42 (0.94–2.15)	1.04 (0.76–1.43)	0.98 (0.84–1.14)	1.08 (1.00–1.17)
Västernorrlands län	1.00 (0.80–1.25)	1.16 (0.69–1.95)	0.71 (0.44–1.15)	0.80 (0.66–0.98)	1.10 (1.01–1.19)
Jämtlands län	1.09 (0.81–1.47)	1.47 (0.80–2.68)	1.19 (0.76–1.84)	0.57 (0.38–0.86)	0.99 (0.86–1.13)
Västerbottens län	0.99 (0.78–1.25)	1.02 (0.56–1.85)	1.21 (0.89–1.66)	0.83 (0.68–1.01)	0.98 (0.89–1.08)
Norrbottens län	1.19 (0.98–1.44)	1.01 (0.57–1.81)	0.86 (0.57–1.28)	0.91 (0.76–1.08)	1.02 (0.93–1.12)
	Finish regions (university hospital regions)				
Tampere Region	1	1	1	1	1
Helsinki Region	0.98 (0.82–1.18)	0.83 (0.54–1.29)	0.94 (0.65–1.37)	1.02 (0.90–1.16)	1.02 (0.93–1.11)
Kuopio Region	1.05 (0.86–1.29)	1.05 (0.66–1.69)	1.15 (0.77–1.74)	1.01 (0.89–1.17)	0.91 (0.93–1.01)
Turku Region	0.86 (0.70–1.07)	1.01 (0.62–1.64)	1.18 (0.77–1.80)	0.93 (0.80–1.08)	0.95 (0.90–1.10)
Oulu Region	0.91 (0.72–1.14)	0.91 (0.53–1.54)	0.92 (0.58–1.46)	1.00 (0.85–1.17)	0.97 (0.93–1.11)

Note: Model 1—Adjusted for age (linear) and sex.

 © 2023. The Author(s).

Supplementary Table S16 Adjusted relative risks (95% CI) 1 year after baseline for the clinical outcomes in patients with atrial fibrillation by region in Denmark, Sweden, and Finland: adjusted as Model 2

	Ischemic stroke	Intracerebral hemorrhage	Intracranial bleeding	Other major bleeding	Total mortality
	Relative risk (95% CI)				
	Danish regions				
Capital	1	1	1	1	1
North	0.65 (0.53–0.81)	1.04 (0.60–1.79)	1.17 (0.74–1.85)	0.99 (0.87–1.13)	1.00 (0.92–1.09)
Central	0.75 (0.64–0.88)	1.47 (0.95–2.27)	1.30 (0.90–1.89)	1.03 (0.92–1.14)	0.93 (0.87–1.00)
South	0.75 (0.64–0.88)	1.00 (0.66–1.53)	0.92 (0.62–1.34)	0.98 (0.88–1.09)	0.91 (0.85–0.98)
Zealand	1.06 (0.91–1.24)	0.93 (0.57–1.52)	0.86 (0.55–1.34)	1.06 (0.95–1.19)	1.14 (1.06–1.22)
	Swedish regions				
Stockholms län	1	1	1	1	1
Uppsala län	0.90 (0.71–1.15)	0.86 (0.62–1.18)	0.50 (0.34–0.73)	0.82 (0.68–0.99)	0.99 (0.91–1.09)
Södermanlands län	1.04 (0.84–1.29)	0.40 (0.20–0.78)	0.57 (0.41–0.80)	0.92 (0.78–1.09)	1.09 (1.00–1.19)
Östergötlands län	1.00 (0.84–1.20)	1.36 (1.08–1.72)	0.85 (0.67–1.06)	0.88 (0.76–1.01)	1.04 (0.97–1.12)
Jönköpings län	0.96 (0.78–1.17)	0.23 (0.10–0.54)	0.29 (0.18–0.48)	0.89 (0.77–1.04)	1.09 (1.01–1.17)
Kronobergs län	0.73 (0.51–1.03)	0.21 (0.04–1.18)	0.27 (0.11–0.68)	1.01 (0.83–1.21)	1.09 (0.99–1.21)
Kalmar län	0.92 (0.73–1.16)	1.11 (0.87–1.41)	0.68 (0.52–0.89)	0.95 (0.81–1.11)	1.17 (1.08–1.26)
Gotlands län	0.65 (0.35–1.24)	0.29 (0.05–1.78)	1.03 (0.71–1.49)	1.01 (0.75–1.35)	0.96 (0.80–1.15)
Blekinge län	1.01 (0.78–1.32)	1.87 (1.49–2.35)	1.53 (1.27–1.85)	0.86 (0.69–1.07)	1.07 (0.97–1.19)
Skåne län	0.95 (0.83–1.09)	0.50 (0.37–0.67)	0.46 (0.37–0.57)	0.98 (0.90–1.07)	1.11 (1.06–1.17)
Hallands län	0.79 (0.62–1.02)	0.51 (0.29–0.90)	0.34 (0.20–0.60)	1.13 (1.00–1.28)	1.13 (1.05–1.22)
Västra Götalands län	0.97 (0.86–1.10)	0.76 (0.63–0.91)	0.55 (0.46–0.65)	0.84 (0.77–0.92)	1.03 (0.98–1.09)
Värmlands län	1.12 (0.93–1.35)	1.27 (1.02–1.58)	1.03 (0.85–1.25)	1.17 (1.04–1.33)	1.22 (1.14–1.31)
Örebro län	1.24 (1.02–1.50)	1.61 (1.29–2.00)	0.93 (0.72–1.18)	0.97 (0.82–1.14)	1.11 (1.02–1.21)
Västmanlands län	1.25 (1.03–1.51)	0.37 (0.19–0.73)	0.51 (0.36–0.74)	1.02 (0.88–1.19)	1.16 (1.07–1.26)
Dalarnas län	1.00 (0.81–1.24)	0.96 (0.71–1.28)	0.82 (0.65–1.04)	0.99 (0.85–1.15)	1.09 (1.00–1.18)
Gävleborgs län	1.09 (0.90–1.32)	0.75 (0.54–1.02)	0.56 (0.41–0.76)	1.08 (0.95–1.23)	1.10 (1.02–1.19)
Västernorrlands län	0.97 (0.78–1.22)	0.32 (0.13–0.80)	0.26 (0.13–0.55)	0.86 (0.72–1.03)	1.14 (1.05–1.24)
Jämtlands län	1.02 (0.75–1.38)	0.35 (0.11–1.12)	0.45 (0.23–0.88)	0.71 (0.51–0.97)	1.00 (0.87–1.14)
Västerbottens län	0.96 (0.76–1.21)	1.26 (0.99–1.61)	0.91 (0.72–1.15)	0.85 (0.71–1.02)	0.99 (0.90–1.09)
Norrbottens län	1.13 (0.93–1.38)	1.06 (0.77–1.45)	0.66 (0.48–0.90)	0.92 (0.78–1.08)	1.03 (0.95–1.12)
	Finish regions (university hospital regions)				
Tampere Region	1	1	1	1	1
Helsinki Region	0.97 (0.81–1.17)	0.82 (0.49–1.36)	0.90 (0.59–1.37)	1.03 (0.91–1.19)	1.00 (0.92–1.09)
Kuopio Region	1.05 (0.85–1.30)	1.03 (0.63–1.67)	1.11 (0.67–1.83)	1.06 (0.90–1.24)	0.90 (0.81–1.00)
Turku Region	0.86 (0.69–1.08)	0.98 (0.58–1.65)	1.06 (0.63–1.81)	0.96 (0.82–1.13)	0.98 (0.89–1.09)
Oulu Region	0.91 (0.72–1.15)	0.90 (0.51–1.57)	0.85 (0.48–1.50)	1.02 (0.87–1.21)	0.97 (0.87–1.08)

Notes: Model 2—adjusted for age (linear), sex, modified CHA_2DS_2-VASc (factor), modified HAS-BLED (factor), statins, and antiplatelets.

 © 2023. The Author(s).

References

1 Lip GY, Skjøth F, Rasmussen LH, Larsen TB. Oral anticoagulation, aspirin, or no therapy in patients with nonvalvular AF with 0 or 1 stroke risk factor based on the CHA2DS2-VASc score. J Am Coll Cardiol 2015;65(14):1385–1394

2 Hvidberg MF, Johnsen SP, Glümer C, Petersen KD, Olesen AV, Ehlers L. Catalog of 199 register-based definitions of chronic conditions. Scand J Public Health 2016;44(05):462–479

 © 2023. The Author(s).
